# Supplementary material for: Adenosine 2A Receptor Activation Amplifies Ibrutinib Antiplatelet Effect; Implications in Chronic Lymphocytic Leukemia
Source: Cancers (Basel). 2022 Nov 23;14(23):5750. doi: 10.3390/cancers14235750 (PMC9741389; doi:10.3390/cancers14235750)
Supplement: Supplementary file 1 [file cancers-14-05750-s001.zip › cancers-2018628-supplementary.pdf]

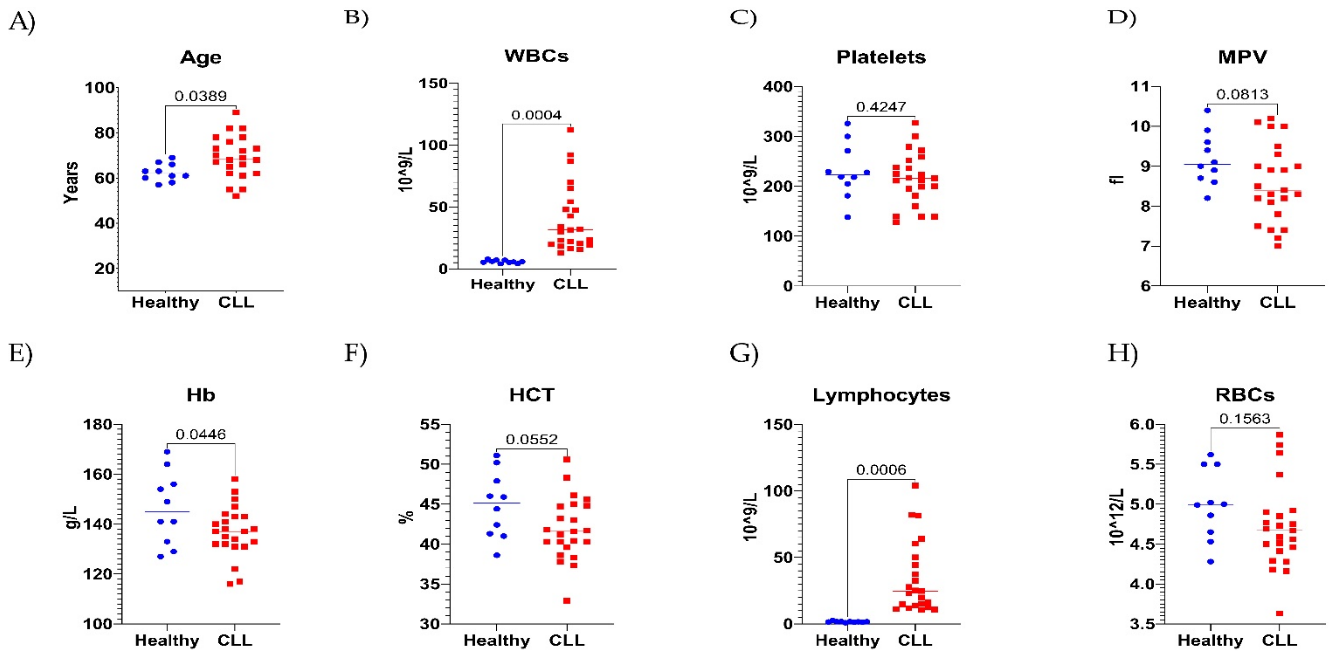

Supplementary Figure S1: Summary of full blood counts in the study subjects

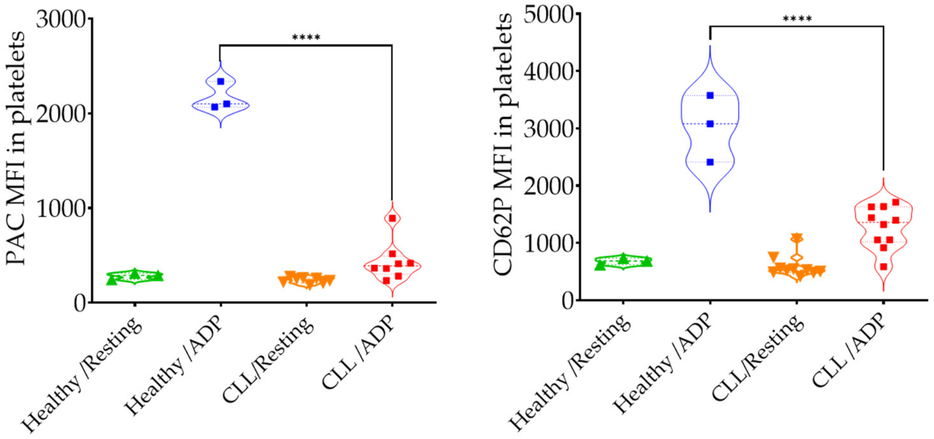

Supplementary Figure S2: Impaired ADP-induced platelet activation in whole blood obtained from CLL patients compared to age-matched healthy volunteers

Blood was collected from healthy volunteers in sodium citrate, then diluted 1:20 in Tyrode-HEPES buffer. Diluted blood was then incubated with ADP (2 µM) or PBS for 15 minutes at 37˚C. The samples were stained with PE-CD62P (P-selectin) and FITC-PAC1 (active-form αIIbβ3) for 15 minutes in the dark, then washed in stain buffer (BD) and fixed in ice-cold 1% PFA in PBS and kept at 4˚C. Samples were examined using BD LSRFortessa™ Flow Cytometer. Platelets were identified by their small size using forward scatter (FSC-A), side scatter (SSC-A) and then plotted between the two activation markers CD62P (Y-axis) and PAC1 (X-axis).

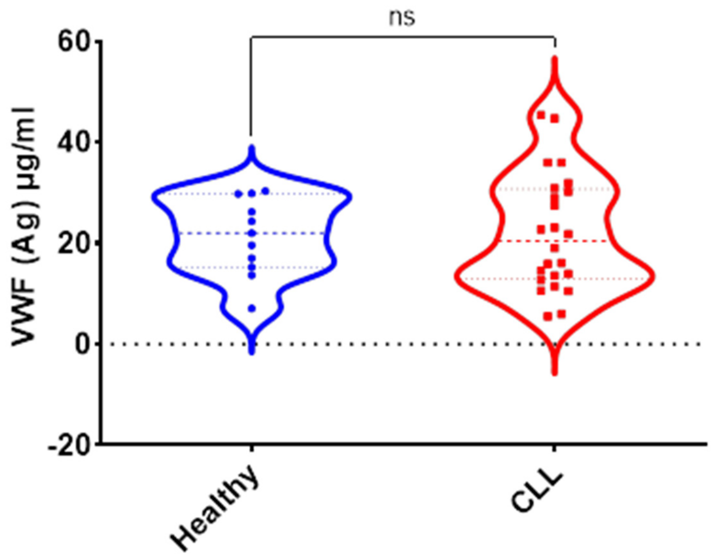

Supplementary Figure S3: Von Willebrand Factor (VWF) in untreated, stable CLL patients compared to age-matched healthy controls. VWF was measured in citrated plasma according using Human Von Willebrand Factor ELISA Kit (ab223864, Abcam) according to the manufacturer's instructions. N=9 (healthy controls) and 23 (CLL)
